# Total Anomalous Systemic Venous Drainage with Heterotaxia Syndrome: A Rare Case

**DOI:** 10.1155/2014/392841

**Published:** 2014-08-12

**Authors:** Ali Yildirim, Pelin Kosger, Gokmen Ozdemir, Birsen Ucar, Zubeyir Kilic

**Affiliations:** Department of Pediatric Cardiology, Medical Faculty, Eskisehir Osmangazi University, 26480 Eskisehir, Turkey

## Abstract

Total anomalous systemic venous return is a very rare anomaly, where vena cava inferior, vena cava superior, and coronary sinus drain into left atrium. Two-day-old male baby was admitted with cyanosis and tachypnea after the birth. Left atrial isomerism with anomalous systemic venous drainage was found on echocardiographic examination. We present an unusual case of total anomalous systemic venous drainage in to the left atrium.

## 1. Introduction

Total anomalous systemic venous return is a very rare anomaly, where vena cava inferior (IVC), vena cava superior (SVC), and coronary sinus drain into left atrium [1]. In this report, we present a case of left atrial isomerism with anomalous systemic venous drainage, in a 2-day-old male patient. There are few published reports regarding the total anomalous systemic venous drainage with heterotaxy syndrome.

## 2. Case Report

**Figure 1:**
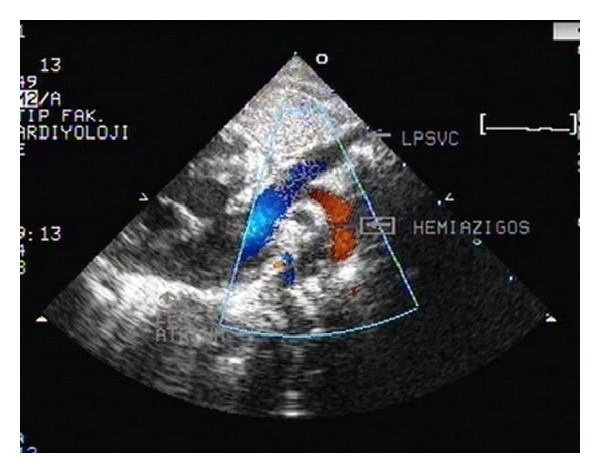
Azygos vein was draining into left SVC.

**Figure 2:**
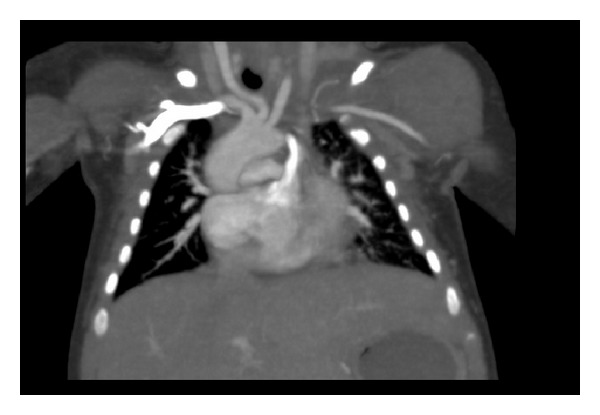
Right subclavian vein flow into left SVC followed by left atrium.

**Figure 3:**
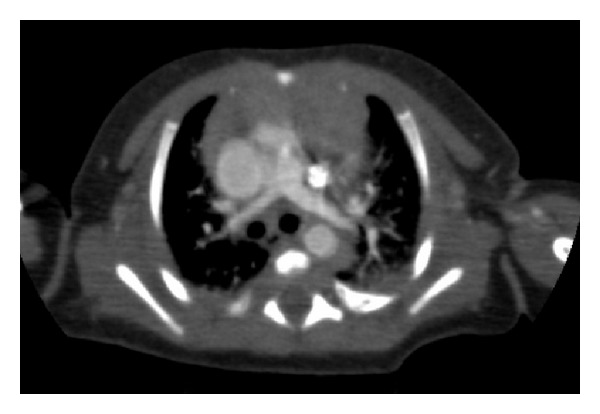
The left SVC is seen in front of the left pulmonary artery.

Physical examination of the baby, who had postnatal cyanosis and tachypnea, was normal. Saturation was 70 percent in room air and cyanosis persisted, although oxygen therapy was started. Electrocardiography and chest X-ray were normal. On echocardiographic examination, the following findings were noted: interrupted IVC was present with left sided azygos continuation (see Supplementary Video  1 available online at http://dx.doi.org/10.1155/2014/392841); azygos vein was draining into left SVC (Figure 1, see Supplementary Video  2); right SVC was absent; and left SVC was draining into left sided atrium via completely unroofed coronary sinus (see Supplementary Video 3). Left cardiac chambers were dilated and nonrestrictive atrial septal defect (ASD) (see Supplementary Video  4) was present. Bridging liver, multiple spleen, and azygos continuation with interruption of the inferior vena cava were seen in abdominal ultrasonography. In catheter laboratory, following administration of contrast agent via left axillary vein (manual) and right subclavian vein (catheter), drainage of both veins into left SVC was followed by left atrium (see Supplementary Video 5). Following administration of contrast agent via left axillary vein (manual) and left hemiazygos vein (catheter), drainage of both veins into left SVC was followed by left atrium (see Supplementary Video 6). Ventricular septal defect (VSD), atrioventricular septal defect wasn't found with echocardiography (see Supplementary Video  7). Angiography showed that pulmonary venous return was normal. Findings were verified with computed tomography (Figures 2 and 3). Patient was transferred to surgery team. Left sided superior vena caval blood was directed to right atrium with baffle. Oxygen saturation increased from 95% to 98%. The patient was discharged on postoperative day 7.

## 3. Discussion

Total anomalous systemic venous return cases are scarce in the literature [2]. This disorder is usually associated with atrioventricular canal defect, common atrium, ASD, VSD, and heterotaxy syndrome [3]. Left-to-right shunt is obligatory for life via ASD, VSD, or patent ductus arteriosus (PDA). Patients experience cyanosis, and paradoxical embolism may be seen. Secondary findings of cyanosis can be seen, such as polycythemia and clubbing, depending on age at diagnosis. Contrast echocardiography is very useful for diagnosis. Patients are divided into two groups depending on interruption of IVC, with respect to intraoperative venous cannulation. In type 1 patients who are characterized with IVC directly drain into left atrium. In type 2 patients have interrupted IVC and it is drained into SVC via azygos system.

## Supplementary Material

It was shown that interrupted IVC was present with left sided azygos continuation at Video 1 and azygos vein was draining into left SVC at video 2. Video 3 showed that left SVC was draining into left sided atrium via completely unroofed coronary sinus. Non-restrictive atrial septal defect was shown at Video 4. Video 5 shown that following administration of contrast agent via left axillary vein (manual) and right subclavian vein (catheter), drainage of both veins into left SVC followed by left atrium. Video 6 shown that following administration of contrast agent via left axillary vein (manual) and left hemiazygos vein (catheter), drainage of both veins into left SVC was followed by left atrium. Video 7 shown that interventricular septum was intact.
